# Extracellular Pectinase from a Novel Bacterium* Chryseobacterium indologenes* Strain SD and Its Application in Fruit Juice Clarification

**DOI:** 10.1155/2018/3859752

**Published:** 2018-03-21

**Authors:** Karabi Roy, Sujan Dey, Md. Kamal Uddin, Rasel Barua, Md. Towhid Hossain

**Affiliations:** Department of Microbiology, University of Chittagong, Chittagong 4331, Bangladesh

## Abstract

Pectinase is one of the important enzymes of industrial sectors. Presently, most of the pectinases are of plant origin but there are only a few reports on bacterial pectinases. The aim of the present study was to isolate a novel and potential pectinase producing bacterium as well as optimization of its various parameters for maximum enzyme production. A total of forty bacterial isolates were isolated from vegetable dump waste soil using standard plate count methods. Primary screening was done by hydrolysis of pectin. Pectinase activity was determined by measuring the increase in reducing sugar formed by the enzymatic hydrolysis of pectin. Among the bacterial isolates, the isolate K6 exhibited higher pectinase activity in broth medium and was selected for further studies. The selected bacterial isolate K6 was identified as* Chryseobacterium indologenes *strain SD. The isolate was found to produce maximum pectinase at 37°C with pH 7.5 upon incubation for 72 hours, while cultured in production medium containing citrus pectin and yeast extract as C and N sources, respectively. During enzyme-substrate reaction phase, the enzyme exhibited its best activity at pH of 8.0 and temperature of 40°C using citrus pectin as substrate. The pectinase of the isolate showed potentiality on different types of fruit juice clarification.

## 1. Introduction

Enzymes are biological molecules that accelerate biochemical reactions. Pectinases are the group of enzymes that prompt the degradation of pectic substances through depolymerization and deesterification reaction [1]. Pectinase is also a well-known term for commercial enzyme preparation during fruit juice clarification. This enzyme disunites polygalacturonic acid into mono-galacturonic acid by opening glycosidic linkages [2].

In the world market, it has been reported that pectinase accounts for 10% of global industrial enzymes produced [3]. Pectinolytic enzymes are produced by many organisms like bacteria, fungi, yeasts, insects, nematodes, protozoan, and plants. Among the various pectinases, bacterial pectinases take more advantages over other pectinases. With the passage of time, many reports have been published on the optimization of different microbiological parameters and fermentation strategies for the production of pectinases [4].

Pectinases have immense applications in fruit juice industries to improve fruit juice clarity and yield [5]. Pectinases also have other various industrial application﻿s like scouring of cotton, degumming of plant fibers, waste water treatment, and vegetable oil extraction so used in various industries as pulp industry, textile industry, food industry, and so on. The application of pectinase enzyme to alter the texture or flavor of fruit juice, to increase extraction and clarification, and to reduce viscosity has also been described [6].

Keeping all the above advantages of pectinase enzymes in consideration, the aim of the present study was designed to isolate potential pectinolytic microorganisms, optimize their cultural conditions for maximum pectinase production, and investigate different factors involved in maximum pectinase activity and also to evaluate its potentiality in different fruit juice clarification.

## 2. Material and Methods

### 2.1. Sampling and Screening

Pectinase producing bacteria were isolated from vegetable dump waste soil. From the collected samples, 40 bacterial isolates were isolated and purified by following standard plate count techniques described by Dubey and Maheshwari [7]. Among 40 isolates, eight isolates were found to produce pectinase while grown on yeast extract pectin agar (YEP) medium during primary screening. Screening of pectinase producing bacteria was carried out in YEP agar medium containing yeast extract 1%, pectin 1%, agar 1.5%, and NaCl 0.5% (pH 7.0) at 37°C for 48 hours of incubation. After incubation, the colonies showing clear zones upon flooding with iodine-potassium iodide solution (1.0 g iodine, 5.0 g potassium iodide, and 330 mL H_2_O) were selected as pectinase producers [8] and the isolate K6 with the maximum zone of diameter was preceded for further studies.

### 2.2. Identification of Selected Bacterial Strain by Phenotypic and Biochemical Characteristics

The selected isolate was identified by morphological, cultural, and biochemical characteristics. Colony characteristics of the isolate were determined on yeast extract-pectin agar slant and cellular morphology by Gram staining method. For biochemical characteristics, several tests like citrate test, TSI (triple sugar iron) test, indole test, MR-VP, motility, catalase, oxidase, starch hydrolysis, and fermentation tests for various sugars such as glucose, sucrose, lactose, maltose, starch, and mannitol tests were done.

### 2.3. Molecular Identification of the Bacterial Isolate Using 16S rRNA Sequencing

Simultaneously, this potential isolate was further identified using the molecular tool of 16S rRNA sequencing. In this method, Promega® Wizard® DNA purification kit was used for the extraction of genomic DNA of the selected isolate. 16S rRNA gene region was amplified with the universal primers. The reaction mixtures were 5 *μ*l of template, primers: 1 *μ*l of forward primer: 27F (5′ AGAGTTTGATCCTGGCTCAG 3′), 1 *μ*l of reverse primer: 1492R (5′ TACCTTGTTACGACTT 3′), 6 *μ*l of assay buffer, 2 *μ*l of Taq DNA polymerase, and 5 *μ*l of dNTP mix. PCR products were purified by using the PCR KlenzolTM and it was sequenced with a next-generation DNA sequencing. The sequencing results were then processed using BioEdit software. The nucleotide sequence analysis was done using the Basic Local Alignment Search Tool (BLAST) program on National Center for Biotechnology Information site (https://www.ncbi.nlm.nih.gov). The obtained sequence data were submitted to NCBI GenBank with accession number KY684254. Phylogenetic analysis was conducted in Molecular Evolutionary Genetics Analysis software version 7.0 (MEGA7) [9].

### 2.4. Preparation of Inoculum

For the production of pectinase enzyme, the inoculum was prepared by inoculating 10 mL of sterilized YEP liquid medium in a test tube with one loop full of pure culture and incubated at 125 rpm at 37°C. The fresh overnight grown pure culture was used as an inoculum for enhanced enzyme production.

### 2.5. Production of Pectinase Enzyme

The inoculum (5% v/v) was transferred aseptically to 50 mL of production medium (yeast extract pectate broth showing the following composition: yeast extract 1%, pectin 1%, NaCl 0.5%, and distilled water 100 ml) in 250 mL conical flask and incubated at 37°C for 72 hours with 125 rpm in a shaking incubator [10]. After incubation, the production medium was centrifuged at 7000 rpm for 15 min at 4°C to obtain cell-free supernatant. The supernatant was used as the crude enzyme for further studies.

### 2.6. Pectinase Assay

The pectinase activity was assayed by estimating the amount of reducing sugars released under assay conditions by the enzymatic degradation of citrus pectin. The reaction mixture containing 1.8 ml substrate (citrus pectin) solution and 0.2 ml suitably diluted enzyme solution was incubated at 40°C in water bath for 1 hour. The amount of reducing sugar liberated was quantified by Nelson's modification of Somogyi method [11, 12]. One unit of enzyme activity (U) was defined as the amount of enzyme required to release 1 *μ*mol of reducing sugar per ml per minute under standard assay conditions (1 U = 1 *μ*molmin^−1^mL^−1^) [13]. The color intensity was measured at 500 nm in a colorimeter [SpectroT60 (UV-VIS RS)] and compared with a standard curve prepared with “D-glucose” (25–200 micrograms). Control was maintained with uninoculated media and boiled enzyme. Relative activity of the enzyme was calculated as the percentage by using the following formula:

(1) Relative  Activity=Activity  of  the  sample×100Maximum  activity  of  the  sample

### 2.7. Total Protein Estimation

The total protein content was determined by Lowry method [14] measuring the absorbance at 600 nm and compared with a standard curve prepared by bovine serum albumin (BSA).

### 2.8. Optimization of Different Factors Involved in Maximum Pectinase Production

#### 2.8.1. Effect of Temperature, pH, and Incubation Time

The bacterial isolate was subjected to different culture conditions to derive the optimum conditions for maximum pectinase production. Pectinase production was estimated at different temperatures (27, 30, 37, 40, and 45°C), a wide range of pH (5.0, 5.5, 6.0, 6.5, 7.0, 7.5, 8.0, 8.5, and 9.0), and different incubation periods (24, 48, 72, 96, and 120 hours), and then the enzyme activity was measured.

#### 2.8.2. Effect of Carbon and Nitrogen Sources

To study the effect of carbon and nitrogen sources on pectinase production from the selected isolate, various carbon sources (citrus pectin, glucose, sucrose, and starch) and nitrogen sources (peptone, yeast extract, ammonium chloride, and potassium nitrate) were supplemented into production medium at a concentration of 0.5% w/v and then the pectinase activity was assayed.

### 2.9. Optimization of Reaction Parameters for the Maximum Pectinase Activity

Various reaction parameters were studied to determine the optimum conditions of the crude pectinase activity at different temperature, pH, reaction time, and substrate concentration.

#### 2.9.1. Effect of Temperature and pH

The optimum temperature and pH of the pectinase enzyme were determined by incubating the reaction mixture having pH 7.5 at various temperatures (30, 37, 40, and 45°C), and then a range of pH (6.0, 6.5, 7.0, 7.5, 8.0, 8.5, and 9.0) at 40°C for 1 hour using different buffers, respectively. For this purpose, substrate (1% w/v citrus pectin) was prepared at different pH values (pH 6.0–9.0) using 0.2 M citrate-phosphate buffer (pH 6.0–7.5) and 0.2 M Tris-HCl buffer (pH 8.0–9.0). Then the pectinase activity was assayed by using the standard assay method.

#### 2.9.2. Effect of Reaction Time

For determination of optimum reaction time, the enzyme stability was studied by incubating the reaction mixture for various time intervals at 10 min, 20 min, 30 min, 40 min, 50 min, and 60 min under optimized temperature and pH and then performing the assay for pectinase activity.

#### 2.9.3. Effect of Substrate Concentration

To examine the effect of substrate concentration during enzyme-substrate reaction, varying concentrations of citrus pectin (0.5, 1.0, 1.5, 2.0, 2.5, and 3.0%) were prepared and then enzyme activity was measured.

### 2.10. Crude Pectinase Enzyme in Fruit Juice Clarification

To observe the effect of crude pectinase on different fruit juice clarification, tubes were labeled as treatment and control. 10 ml of crude pectinase was taken into the test tube (treatment) and 10 ml of distilled water was taken into the control test tube. Then twenty ml of apple/grape juice was added to both tubes. The contents of the tubes were agitated to mix the enzymes throughout the juice. The tubes were kept into the water bath at 40°C. The tubes were observed at 5-minute intervals over one-hour period. After incubation, the solution was filtered [15].

## 3. Results and Discussion

**Figure 1:**
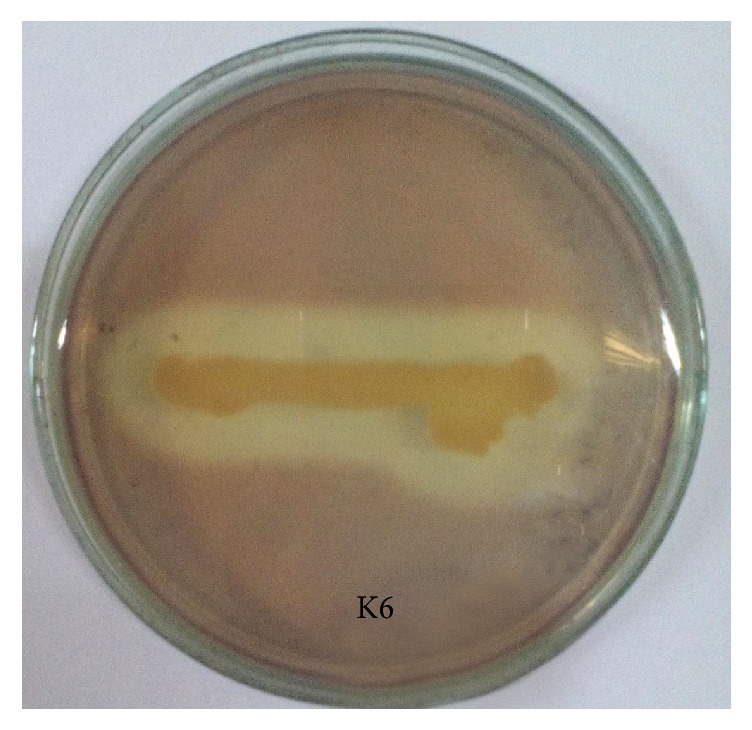
Zone of pectin hydrolysis on YEP agar medium of isolate K6 after 48-hour incubation.

There are only a few reports on bacterial pectinases till now [16]. In this study, we aimed to find out novel and potential pectinolytic bacteria, to optimize the cultural conditions for enhancing enzyme production and partial characterization of the enzyme. For this purpose, we collected vegetable dump waste soil as the source of potential organisms. Eight isolates were primarily selected from 40 bacterial isolates on the basis of their pectinolytic activity, as shown in Figure 1, during screening and finally, the isolate K6 was selected for further studies.

### 3.1. Identification of Selected Bacterial Isolate

To identify the selected isolate K6, both traditional microbiological methods and modern molecular technologies were considered. On the basis of observed morphological, cultural, and biochemical characteristics, the selected isolate was compared with the standard description in Bergey's Manual of Determinative Bacteriology [17] and the isolate K6 was provisionally identified as* Chryseobacterium indologenes*.

**Figure 2:**
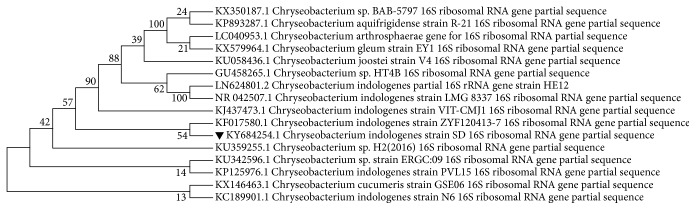
Phylogenetic tree constructed on the basis of 16S rRNA gene sequences of* Chryseobacterium indologenes* strain SD with other* Chryseobacterium* sp. obtained from GenBank database. Their names and respective accession numbers are shown on the tree.

In NCBI database, BLAST showed significant alignments of* Chryseobacterium indologenes* with 95% similarities. Further, the obtained sequence was compared with other related sequences to find the closest homolog at NCBI using BLAST. The construction of a phylogenetic tree (Figure 2) was made on the basis of 16S rRNA gene sequences of isolate* Chryseobacterium indologenes* strain SD and other strains of* Chryseobacterium* species obtained from GenBank database. The related 16 nucleotide sequences were used to construct the phylogenetic tree using the neighbor-joining method [18]. The bootstrap consensus tree inferred from 1000 replicates [19] is taken to represent the evolutionary history of the taxa analyzed [19]. Branches corresponding to partitions reproduced in less than 50% bootstrap replicates are collapsed. The percentage of replicate trees in which the associated taxa clustered together in the bootstrap test (1000 replicates) is shown next to the branches [19]. The evolutionary distances were computed using the Maximum Composite Likelihood method [20] and are in the units of the number of base substitutions per site. The analysis involved 16 nucleotide sequences. All positions containing gaps and missing data were eliminated. There were a total of 1323 positions in the final dataset. Evolutionary analyses were conducted in MEGA7 [9]. Finally, the result attained from the 16S rRNA analyses inferred that the* Chryseobacterium indologenes* strain SD was very close to other* Chryseobacterium indologenes* strains.

### 3.2. Optimization Studies of the Isolate for Pectinase Production

To optimize the culture conditions of this potential organism in the production medium different temperature, pH, incubation period, and various C and N sources were considered. Enzyme production went up with the increase of temperature up to 37°C and then declined (Table 1). The maximum production which occurred at this temperature was 0.679 U/ml (100% of relative activity). This dramatically reduced to nearly 27% at 45°C temperature. In the previous study of Aaisha and Barate (2016) [21], the highest pectinase production was observed from some* Bacillus species* at 37°C which is similar to our current study. The same findings were also reported by the other workers [22] in case of mutagenic strain of* Leuconostoc mesenteroides.*

The same trend was also observed when this organism was allowed to grow in the production medium at varying pH (Table 2). Maximum production (0.564 U/ml) was recorded at pH 7.5. This finding is in accordance with other workers who reported that most of the* Bacillus *sp. produce high amount of pectinase between pH 7.5 and 8 [21, 23]. At highly acidic and alkaline pH, enzyme production decreased by almost 34% and 64%, respectively. From this result, it can be inferred that, at very low and very high pH condition, growth of the organism slows down.

An attempt was made to determine the most favorable time period for enzyme production by the selected isolate and the highest enzyme production (0.594 U/ml) was recorded at 72 hours of incubation (Table 3). The enzyme production gradually decreased to 0.35 *μ*molmin^−1^mL^−1^ at 120 hours of incubation which is almost 40% less than that of maximum. This might be due to the accumulation of waste products at prolonged incubation time with limited nutrient sources which consequently suppressed the growth of microorganism. According to Nawawi et al. (2017) [24], maximum pectinase production was determined from the* Bacillus subtilis* ADI1 after 72 hours of incubation which well agreed with our findings.

In this study, we also supplemented different types of carbon and nitrogen sources to find out the suitable production medium for pectinase production by* Chryseobacterium indologenes *strain SD. Among the four C sources, the organism lost almost 50% of production in case of sucrose utilization. It showed the highest production of 0.671 U/ml, while citrus pectin was used in the medium as C source and almost the same result was found in case of glucose supplement (Table 4) suggesting that the organism exploited citrus pectin more efficiently as compared to other C sources. Prakash et al. (2014) [25] observed the highest production of pectinase with lactose and glucose and Jayani et al. (2010) [26] reported citrus pectin as the best carbon source for pectinase production by* Bacillus sphaericus. *However, some researchers reported the maximum pectinase production from* Bacillus subtilis* ADI1 using rice brain as carbon source [24].

Likewise, the best enzyme production of this isolate was recorded when yeast extract was used as N source in the medium and the organism also produced nearly the same amount of enzyme when peptone was supplemented which indicates that this organism preferred yeast extract as compared to other N sources (Table 5). On top of that, our study revealed that organic nitrogen was used as better N sources by this organism than inorganic sources for enzyme production. These results are completely aligned with the findings of the other workers [25, 26].

Finally, by applying all the optimized parameters, the isolate was allowed to produce the enzyme in the production medium and we observed little increase in pectinase production (0.689 U/ml).

### 3.3. Total Protein Estimation for Crude Enzyme

Protein concentration of the crude enzyme was determined by Folin-Lowry method [14]. The total protein content was 1320 *μ*g/ml in the cell-free supernatant of* Chryseobacterium indologenes *strain SD.

### 3.4. Optimization Studies of Pectinase Activity

By growing the organism in the production medium under optimized conditions, the crude was collected by centrifugation to determine optimum conditions of the pectinase activity. Then the collected crude enzyme was allowed to react with different substrate concentrations (citrus pectin) at a wide range of temperatures, pH, and reaction time.

**Figure 3:**
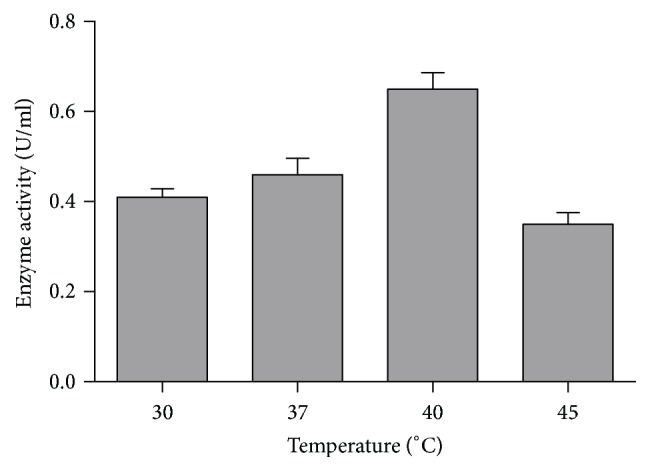
Effect of temperature on pectinase activity.

In this research work, the crude enzyme obtained from* Chryseobacterium indologenes* strain SD showed maximum activity at 40°C (Figure 3), whereas the organism showed the highest production at 37°C. This result is approximately similar to the result of other studies [27]. However, some studies reported [28] that pectinases from various* Bacillus species *were most active at 50°C and 60°C. From our study, it can be inferred that pectinase enzyme produced by the isolate is a moderately thermophilic enzyme.

**Figure 4:**
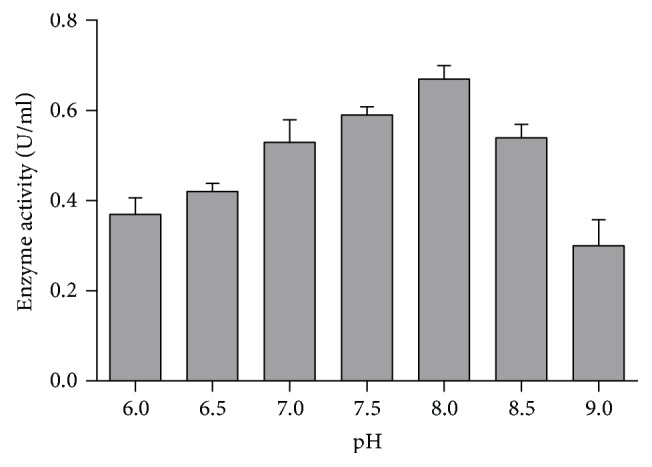
Effect of pH on pectinase activity.

Enzyme activity also depends on the pH of the reaction mixture. In our study, crude enzyme showed the highest activity at slightly alkaline pH 8 (Figure 4) and the organism also showed its maximum production at pH 7.5. Therefore, this enzyme can be used for vegetable purees and other preparations which need neutral to slightly alkaline pH [29]. This finding is in accordance with the reports of previous studies [28]. So, the result of our study indicates that this crude enzyme might be alkaline in nature.

**Figure 5:**
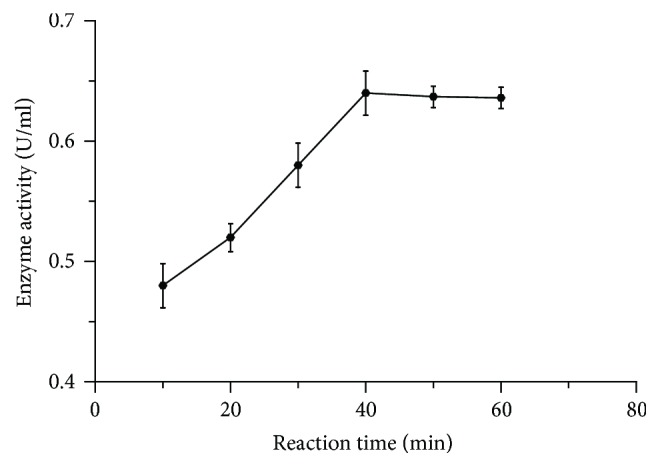
Effect of reaction time on pectinase activity.

As incubation time affects the activity of the enzyme, the crude enzyme was allowed to react with 1% of citrus pectin as substrate at optimized pH and temperature for different time intervals to determine its optimum reaction time. In our study, enzyme activity increased with the increase of incubation time up to 40 min and then remained stable in the subsequent incubation period (Figure 5). This result is in a good agreement with other studies [30].

**Figure 6:**
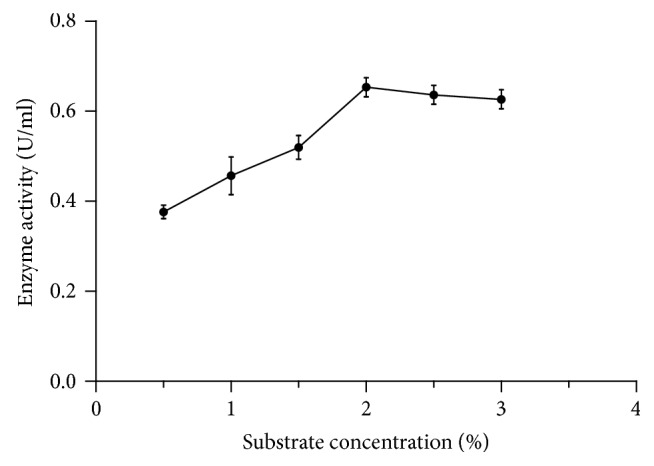
Effect of substrate (citrus pectin) concentration on pectinase activity.

The enzyme assay using different concentrations of substrate (citrus pectin) was observed and found that the enzyme activity augmented up to 2% of pectin and then it showed downward trend before being leveled off at 98% of relative activity in the subsequent increase of substrate concentration (Figure 6). This might be due to complete saturation of enzyme by the substrate.

### 3.5. Application of Crude Pectinase Enzyme in Fruit Juice Clarification

**Figure 7:**
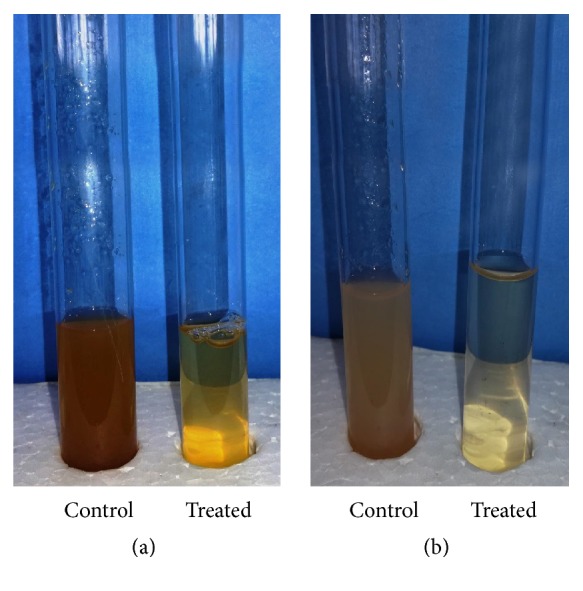
Application of pectinase in apple juice (a) and grape juice (b) clarification.

The effect of crude pectinase of the bacterial isolate* Chryseobacterium indologenes* strain SD was studied for apple/grape juice clarification. The crude enzyme of the selected isolate showed good activities by clarifying the juices as compared to control (Figure 7).

## 4. Conclusion

Nowadays, the need of industrially important enzymes has increased rapidly. Pectinase enzyme has taken great attraction in the field of juice clarification and other commercial applications. In this study,* Chryseobacterium indologenes *strain SD was found as a potential pectinase producer and it is the first report on* Chryseobacterium indologenes *strain SD. In this investigation, the crude enzyme was found to be slightly alkaline in nature and best active at 40°C for 40 minutes of incubation. Further studies can be done for complete characterization and purification of the crude enzyme and purified enzymes can be used in various types of fruit juice clarification.

## Figures and Tables

**Table 1:** Effect of temperature on pectinase production from *Chryseobacterium indologenes* strain SD.

Incubation temperature (°C)	Enzyme activity (*µ*molmin^−1^mL^−1^)	Relative activity (%)
27	0.36 ± 0.050	52.94
30	0.45 ± 0.020	66.18
*37*	*0.68 ± 0.020*	*100.0*
40	0.24 ± 0.021	35.3
45	0.18 ± 0.026	26.47

Values are mean ± SD of 3 replicates.

**Table 2:** Effect of pH on pectinase production from *Chryseobacterium indologenes* strain SD.

pH	Enzyme activity (*µ*molmin^−1^mL^−1^)	Relative activity (%)
5.0	0.37 ± 0.020	66.07
5.5	0.42 ± 0.026	75.00
6.0	0.45 ± 0.026	80.36
6.5	0.47 ± 0.020	83.93
7.0	0.48 ± 0.020	85.71
*7.5*	*0.56 ± 0.021*	*100.0*
8.0	0.40 ± 0.025	71.43
8.5	0.33 ± 0.020	58.93
9.0	0.21 ± 0.038	37.5

Values are mean ± SD of 3 replicates.

**Table 3:** Effect of incubation period on pectinase production from *Chryseobacterium indologenes* strain SD.

Incubation period (hour)	Enzyme activity (*µ*molmin^−1^mL^−1^)	Relative activity (%)
24	0.45 ± 0.020	76.27
48	0.50 ± 0.040	84.75
*72*	*0.59 ± 0.030*	*100.0*
96	0.52 ± 0.021	88.14
120	0.35 ± 0.032	59.32

Values are mean ± SD of 3 replicates.

**Table 4:** Effect of carbon sources on pectinase production from *Chryseobacterium indologenes* strain SD.

Carbon source	Enzyme activity (*µ*molmin^−1^mL^−1^)	Relative activity (%)
*Citrus pectin*	*0.67 ± 0.020*	*100.0*
Glucose	0.64 ± 0.021	95.52
Sucrose	0.38 ± 0.030	56.72
Starch	0.50 ± 0.020	74.63

Values are mean ± SD of 3 replicates.

**Table 5:** Effect of nitrogen sources on pectinase production from *Chryseobacterium indologenes *strain SD.

Nitrogen source	Enzyme activity (*µ*molmin^−1^mL^−1^)	Relative activity (%)
*Yeast extract*	*0.61 ± 0.020*	*100.0*
Peptone	0.55 ± 0.020	90.16
Potassium nitrate	0.46 ± 0.036	75.41
Ammonium chloride	0.42 ± 0.021	68.85

Values are mean ± SD of 3 replicates.
